# Estimated Sleep Duration Before and During the COVID-19 Pandemic in Major Metropolitan Areas on Different Continents: Observational Study of Smartphone App Data

**DOI:** 10.2196/20546

**Published:** 2021-02-02

**Authors:** Rebecca Robbins, Mahmoud Affouf, Matthew D Weaver, Mark É Czeisler, Laura K Barger, Stuart F Quan, Charles A Czeisler

**Affiliations:** 1 Division of Sleep and Circadian Disorders Departments of Medicine and Neurology Brigham and Women's Hospital Boston, MA United States; 2 Division of Sleep Medicine Harvard Medical School Boston, MA United States; 3 Department of Mathematics Kean University Union, NJ United States; 4 School of Psychological Sciences Turner Institute Brain and Mental Health Monash University Victoria Australia; 5 Institute for Breathing and Sleep Austin Health Melbourne Australia

**Keywords:** sleep health, mobile health, sleep tracking, COVID-19, sleep, observational study, app

## Abstract

**Background:**

Amid the COVID-19 pandemic, public health policies to curb the spread of SARS-CoV-2 and its associated disease, COVID-19, have resulted in significant alterations to daily routines (eg, work-from-home policies) that may have enabled longer sleep duration among the general population.

**Objective:**

We aimed to examine changes in estimated sleep duration in 5 major metropolitan areas before and after the start of the COVID-19 pandemic.

**Methods:**

We conducted a prospective observational study using estimated sleep duration data obtained from a smartphone app. The data were obtained from regular users of the smartphone app before and after the World Health Organization declared COVID-19 a pandemic in March 2020. We compared within-subject estimated sleep duration before and during the COVID-19 pandemic using generalized linear mixed models.

**Results:**

Among the 2,871,037 observations, 957,022 (33.3%) were from users in London; 549,151 (19.1%) were from users in Los Angeles; 846,527 (29.5%) were from users in New York City; 251,113 (8.7%) were from users in Seoul; and 267,224 (9.3%) were from users in Stockholm. The average age of the users in the sample was 35 years (SE 11 years). Prior to the COVID-19 pandemic, people residing in Seoul had the shortest estimated sleep duration (mean 6 hours 28 minutes, SE 11.6 minutes) and those residing in Stockholm had the longest estimated sleep duration (mean 7 hours 34 minutes, SE 9.9 minutes). The onset of the COVID-19 pandemic was associated with a 13.7 minute increase in estimated sleep duration when comparing March 2019 and March 2020 (95% CI 13.1-14.3, *P*<.001) and an increase of 22.3 minutes when comparing April 2019 and April 2020 (95% CI 21.5-23.1, *P*<.001).

**Conclusions:**

The average estimated sleep duration increased sharply in the months after the onset of the COVID-19 pandemic. This finding suggests that the implementation of COVID-19 mitigation strategies has provided people worldwide with increased opportunities to sleep, which may enhance the response of the immune system to viral pathogens.

## Introduction

Public health officials worldwide have implemented stringent measures to curb the spread of SARS-CoV-2 and its associated disease, COVID-19. In some regions, actions to mitigate COVID-19 have been drastic, such as mandatory shelter-in-place regulations, while regulations in other regions have been more lenient [1]. In either case, life has changed markedly for much of the global population.

Research conducted amid crises of similar magnitude to the COVID-19 pandemic has shown that sleep is disrupted during and after such events [2-5]. For instance, research conducted after the 2003 severe acute respiratory syndrome (SARS) outbreak in China demonstrated an increase in insomnia symptoms associated with the onset of the outbreak [4]. In the context of a natural disaster, researchers found that 40% of people who survived an earthquake in Japan reported sleep difficulties in the years following the disaster, and 8% reported short sleep duration [6]. Although previous literature would suggest that sleep duration is likely to decline, sleep duration may increase worldwide during the COVID-19 pandemic for several reasons. First, due to the highly contagious nature of SARS-CoV-2 and the lack of a vaccine, social distancing and work-from-home recommendations and policies have been widely implemented to curb the spread of the virus [7]. As much of the global population is spending less time commuting, more time at home, and less time socializing, it is possible that their sleep duration will increase, contrary to what has been observed during previous crises.

Sleep is a critical element of immune system function [8,9]. Experimental studies have shown that inadequate sleep results in increased susceptibility to viral infection [10,11]. Research has also shown a heightened ability to mount an immune response among those who obtain a healthy, sufficient duration of sleep (ie, 7-9 hours) [12-14]. As the COVID-19 pandemic unfolds, surveillance of sleep duration may be important to identify poor sleep practices and to develop evidence-based interventions and campaigns to enhance sleep in response to this crisis as necessary. To further our understanding of sleep during the COVID-19 pandemic, we analyzed smartphone app–estimated sleep durations of individuals residing in London, England; Los Angeles, California, United States; New York City, New York, United States; Seoul, South Korea; and Stockholm, Sweden, before the onset of the COVID-19 pandemic (January 2019 through April 2019) and after the onset of the pandemic (January 2020 to April 2020).

## Methods

### Participants

We conducted a prospective observational study using data obtained from the smartphone-based sleep tracking software app Sleep Cycle. We obtained data from regular users of the app who tracked sleep on 80% or more of the days between January 1, 2019 and April 12, 2020 (at least 374/468 days). To understand geographic variations in sleep during the COVID-19 pandemic, we obtained data from individuals living in 5 major metropolitan areas (London, Los Angeles, New York City, Seoul, and Stockholm).

The selection of geographical regions was guided by several factors. First, urban regions were included in this study because there is a lower density of users in rural regions, which could have hindered comparison between rural and urban regions. Second, there were interesting differences in COVID-19 prevalence, preparedness, and mitigation strategies by geographic region. For instance, Sweden did not limit social mobility among its residents as strictly as other regions, such as the United States. Additionally, South Korea had experience combating a pandemic from the 2003 SARS outbreak. Therefore, we requested data from large urban centers on 3 different continents with the aim of exploring different patterns in estimated sleep duration from country to country that may be reflective in part of different prevalence, preparedness, or mitigation strategies implemented in these various countries.

The Sleep Cycle app logs a participant’s place of residence by the coordinates identified by their smartphone GPS, which participants agree to provide when using the app. Only users who agreed to Sleep Cycle’s privacy policy, which dictates that data may be used for research purposes, were included in the data set.

### Estimated Sleep Duration

Users open the app when they go to bed and first attempt to sleep; they place their smartphone either next to their bed or on their mattress, and they close the app either after the built-in alarm clock wakes them or when they wake naturally from sleep. Sleep is calculated by the app as the time interval between these two digital sleep diary events (first attempt to sleep and waking) [15], in accordance with the 2016 Consumer Technology Association standards for wearable sleep monitors [16], herein termed “estimated sleep duration.” We obtained an anonymized data set that included dated estimated sleep durations for each user between January 1, 2019 and April 12, 2020. The data set comprised 2,974,922 observations. We removed observations with a duration shorter than 1 hour from the data set to mitigate the impact of napping on the estimated sleep duration. These shorter observations represented 3.5% of the sample (103,885/2,974,922 observations). Removal of these shorter observations resulted in a final data set with 2,871,037 observations for analysis.

### Statistical Analyses

Descriptive statistics were used to characterize the demographic characteristics of the sample. To evaluate population-level changes in estimated sleep duration during the COVID-19 pandemic while accounting for established seasonal variation, we compared the estimated sleep duration by geographic location in the same calendar month between years using generalized linear mixed models. This method included hierarchical random effects for the city and study participants, which accounted for the dependence between repeated measures and the clustering of respondents in each location. This design enabled us to compare estimated sleep duration over time before and after the COVID-19 pandemic (eg, March 2019 vs March 2020) in each geographic location. All analyses were performed in Stata Statistical Software for Mac Version 16 (StataCorp LLC).

## Results

In total, 2,871,037 nights from 8218 unique users between January 1, 2019 and April 12, 2020 were available for analysis. Of the 2,871,037 estimated sleep durations analyzed, 957,022 (33.3%) were from London; 549,151 (19.1%) were from Los Angeles; 846,527 (29.5%) were from New York City; 251,113 (8.7%) were from Seoul; and 267,224 (9.3%) were from Stockholm. The average age of users in the sample was 35 years (SE 11 years). Prior to the COVID-19 pandemic, those residing in Seoul had the shortest average estimated sleep duration (mean 6 hours 28 minutes, SE 11.6 minutes) and those residing in Stockholm had the longest average estimated sleep duration (mean 7 hours 34 minutes, SE 9.9 minutes); the average estimated sleep durations were 7 hours 32 minutes (SE 5.5 minutes) in London, 7 hours 21 minutes (SE 8.4 minutes) in Los Angeles and 7 hours 22 minutes (SE 6.3 minutes) in New York City. Table 1 displays descriptive demographic and estimated sleep duration characteristics by city prior to the COVID-19 pandemic.

**Table 1:** Descriptive statistics summarizing the participants’ demographic characteristics and sleep data by city prior to the COVID-19 pandemic (N=2,871,037 nights; N=8,218 users).

Characteristic			Value	95% CI	SE
**London (n=957,022 nights)**					
	Age (years), mean		34.4	34.3-34.4	0.0
	**Gender (n=2687 users), n (%)**				
		Male	1423 (53.5)	51.8-55.6	0.0
		Female	1236 (46.5)	44.4-48.2	0.0
	Estimated sleep duration		7 hours 32 minutes	7 hours 32 minutes–7 hours 33 minutes	5.5 minutes
**Los Angeles (n=549,151 nights)**					
	Age (years), mean		36.0	36.1-36.1	0.02
	**Gender (n=1452 users), n (%)**				
		Male	787 (54.2)	51.6-56.8	0.0
		Female	665 (45.8)	43.2-48.4	0.0
	Estimated sleep duration		7 hours 21 minutes	7 hours 21 minutes–7 hours 22 minutes	8.4 minutes
**New York City (n=846,527 nights)**					
	Age (years), mean		35.0	34.9-35.1	0.0
	**Gender (n=2398 users), n (%)**				
		Male	1209 (50.6)	48.6-52.6	0.0
		Female	1182 (49.4)	47.4-51.4	0.0
	Estimated sleep duration		7 hours 22 minutes	7 hours 22 minutes–7 hours 22 minutes	6.3 minutes
**Seoul (n=251,113 nights)**					
	Age (years), mean		33.8	33.8-33.8	0.0
	**Gender (n=809 users), n (%)**				
		Male	488 (60.3)	56.9-63.6	0.0
		Female	321 (39.7)	36.4-43.1	0.0
	Estimated sleep duration		6 hours 28 minutes	6 hours 28 minutes–6 hours 28 minutes	11.6 minutes
**Stockholm (n=267,224 nights; n=868 users)**					
	Age (years)		40.7	40.7-40.8	0.0
	**Gender**				
		Male	450 (51.8)	48.5-55.2	0.0
		Female	418 (48.2)	44.8-51.5	0.0
	Estimated sleep duration		7 hours 34 minutes	7 hours 33 minutes–7 hours 34 minutes	9.9 minutes

**Figure 1:**
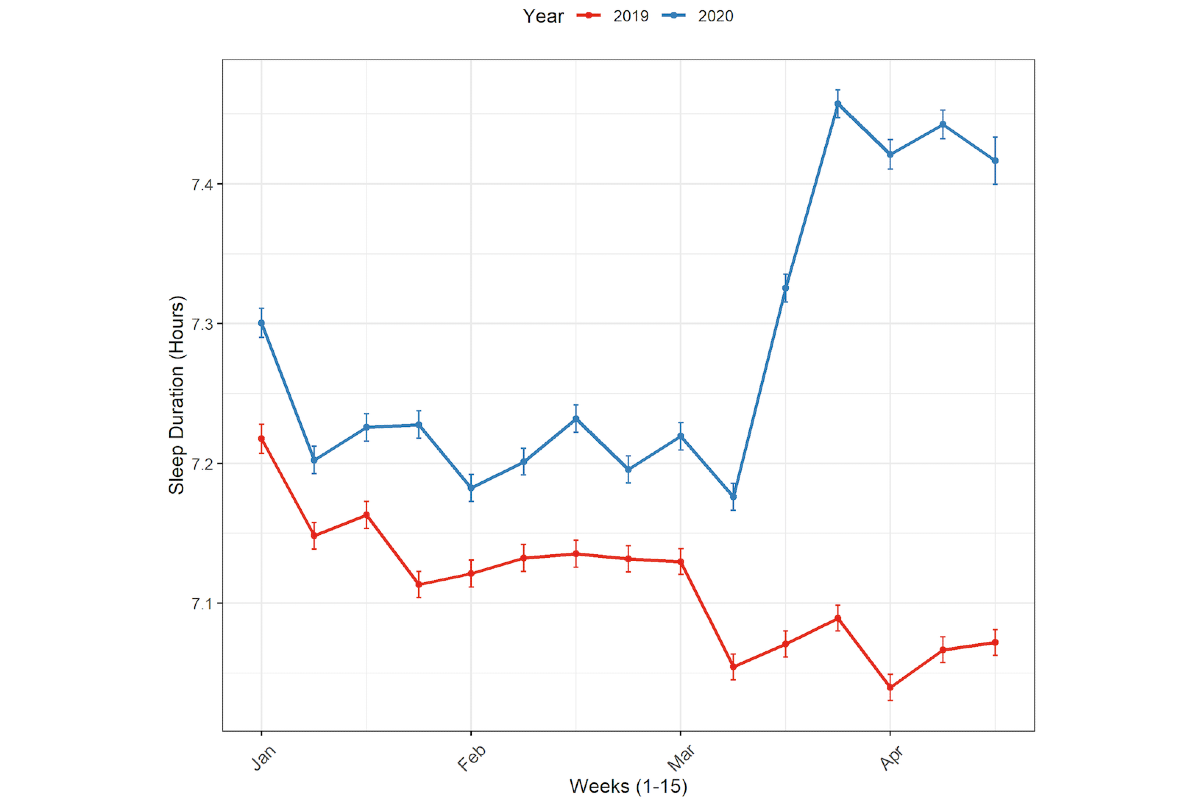
Estimated sleep duration by month (January to April) in 2019 and in 2020 for the total sample. The I-bars represent the 95% CIs.

**Figure 2:**
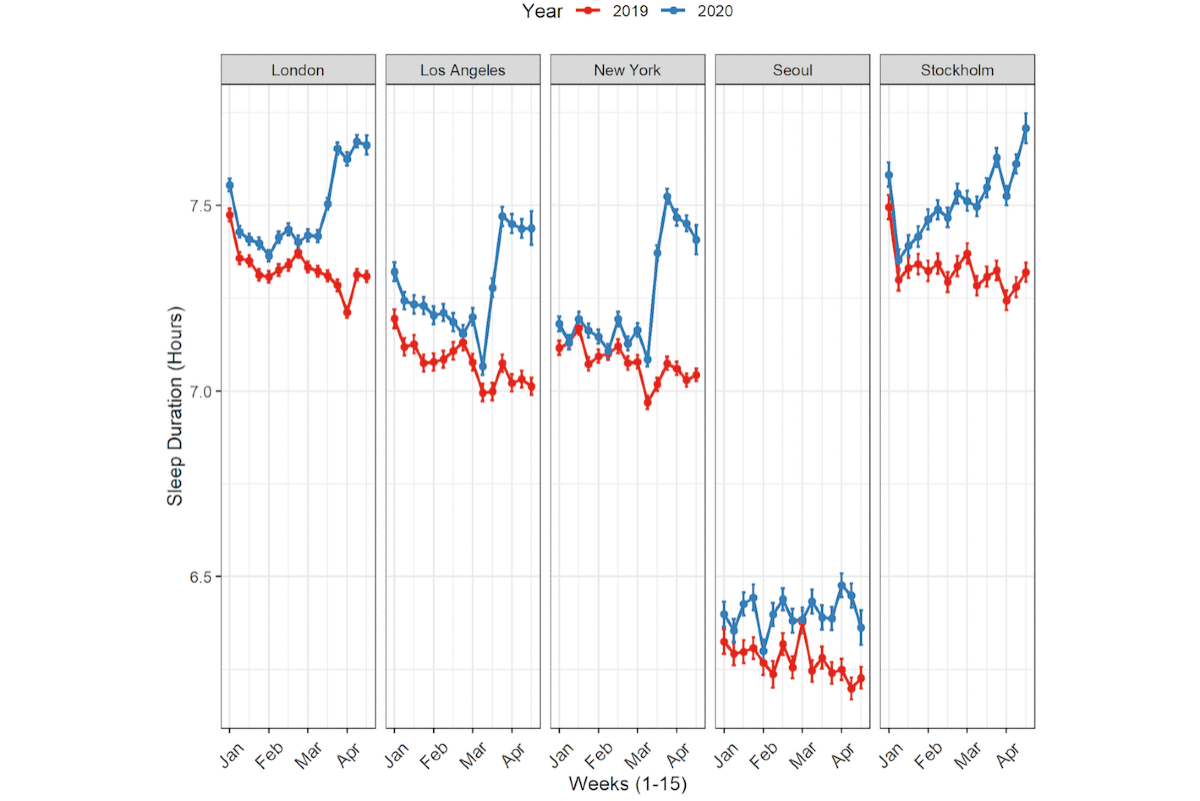
Estimated sleep duration by months (January to April) in 2019 and in 2020 for the total sample by city.

As shown in Figures 1 and 2, there was an increase in estimated sleep duration starting in February 2020 compared to February 2019, which coincided with the onset of the COVID-19 pandemic in the 5 cities across 3 continents.

**Figure 3:**
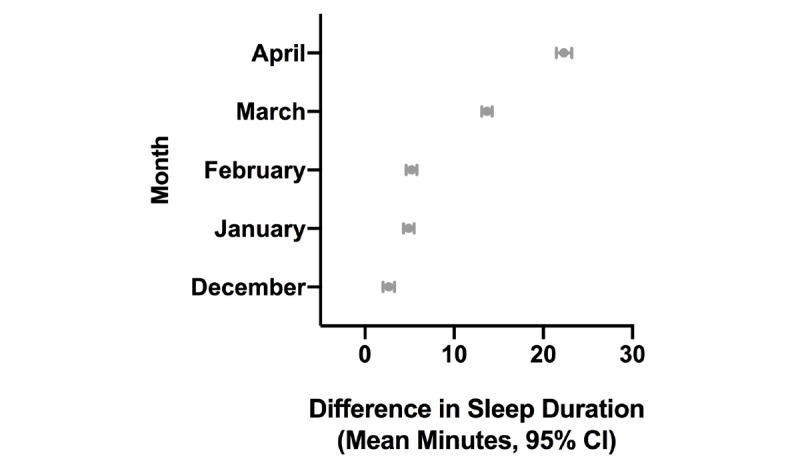
Difference in estimated sleep duration during the COVID-19 pandemic compared to the same month in the previous year in the total sample.

**Figure 4:**
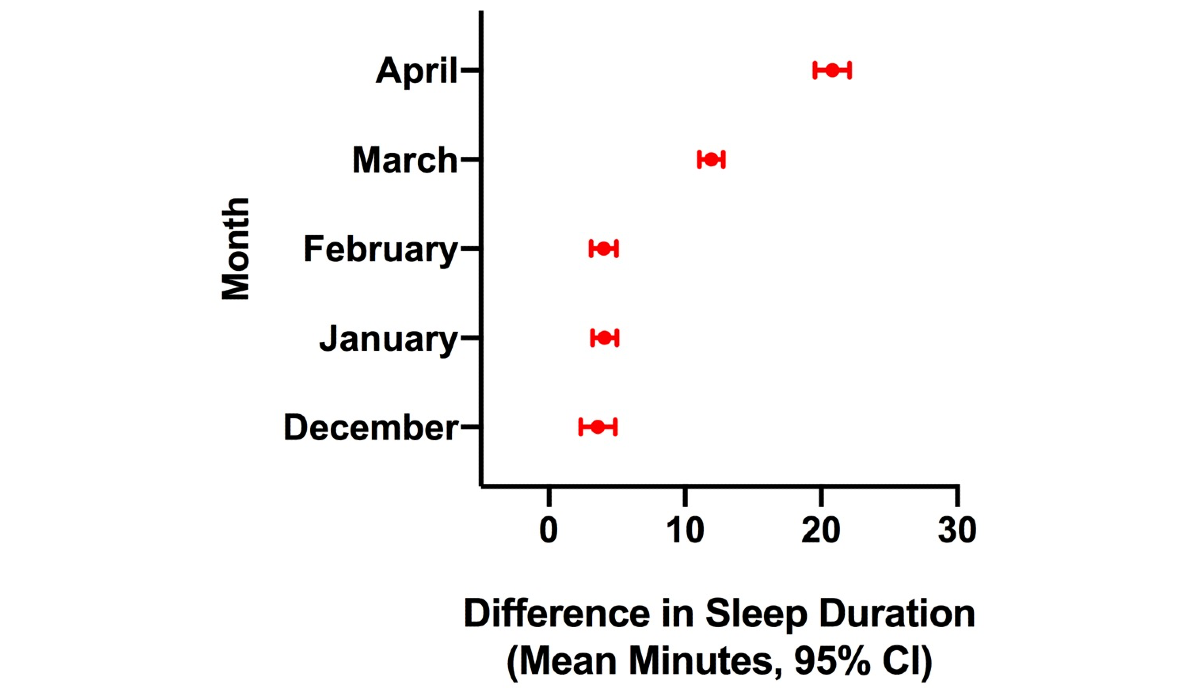
Difference in estimated sleep duration during the COVID-19 pandemic compared to the same month in the previous year in London.

**Figure 5:**
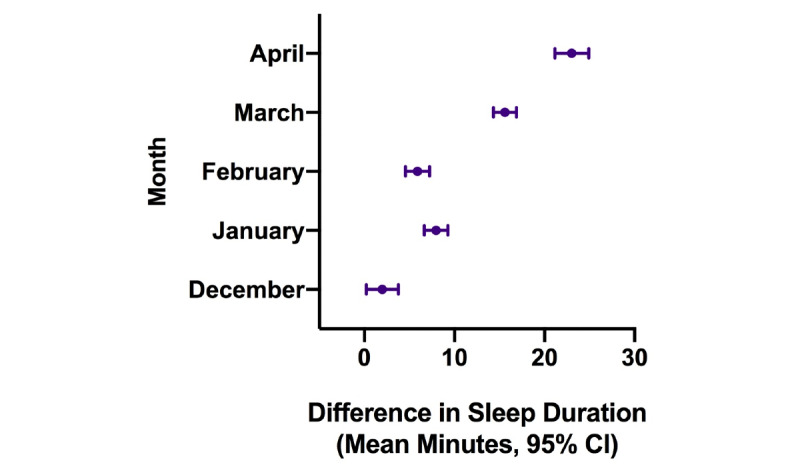
Difference in estimated sleep duration during the COVID-19 pandemic compared to the same month in the previous year in Los Angeles.

**Figure 6:**
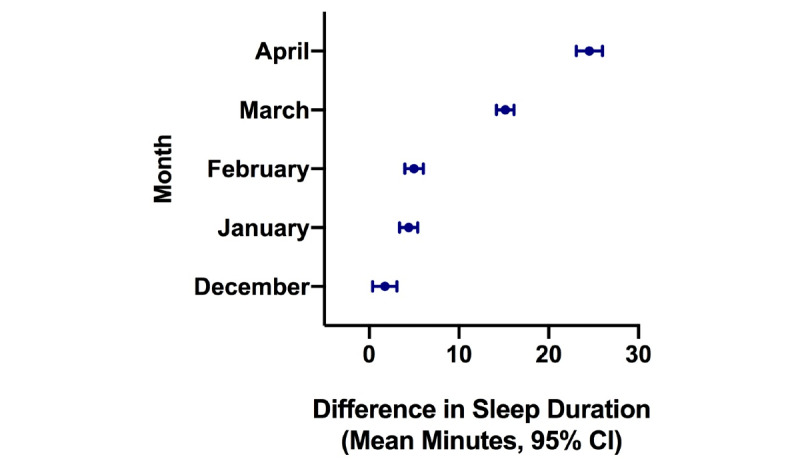
Difference in estimated sleep duration during the COVID-19 pandemic compared to the same month in the previous year in New York.

**Figure 7:**
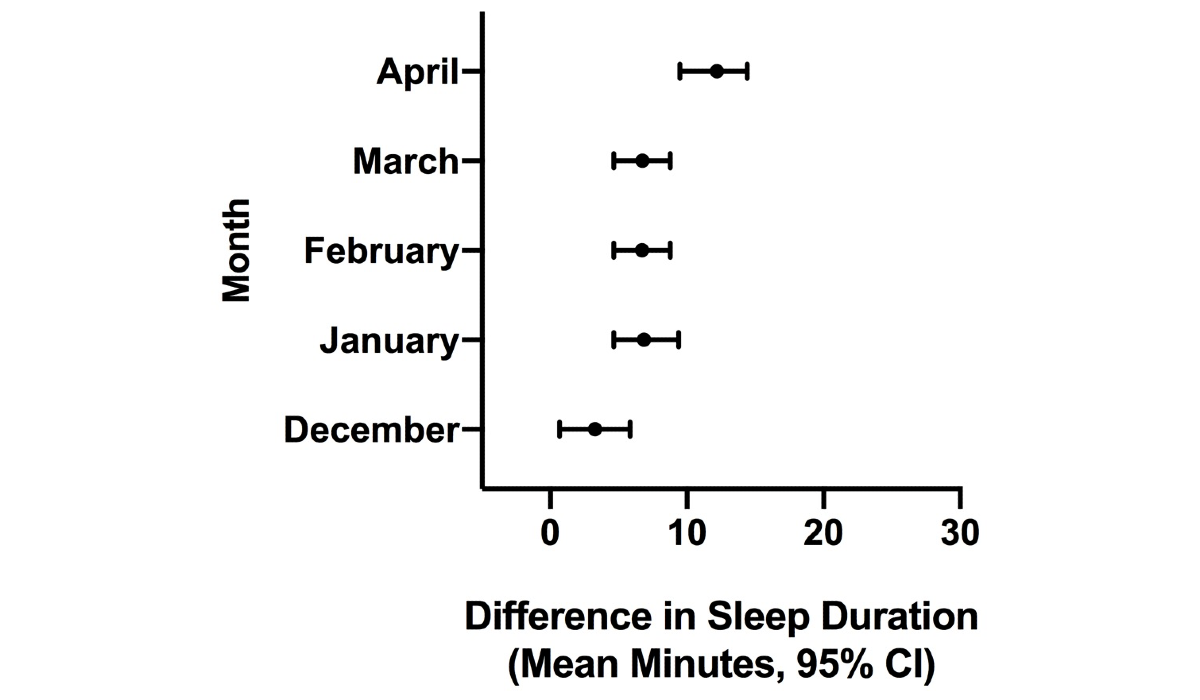
Difference in estimated sleep duration during the COVID-19 pandemic compared to the same month in the previous year in Seoul.

**Figure 8:**
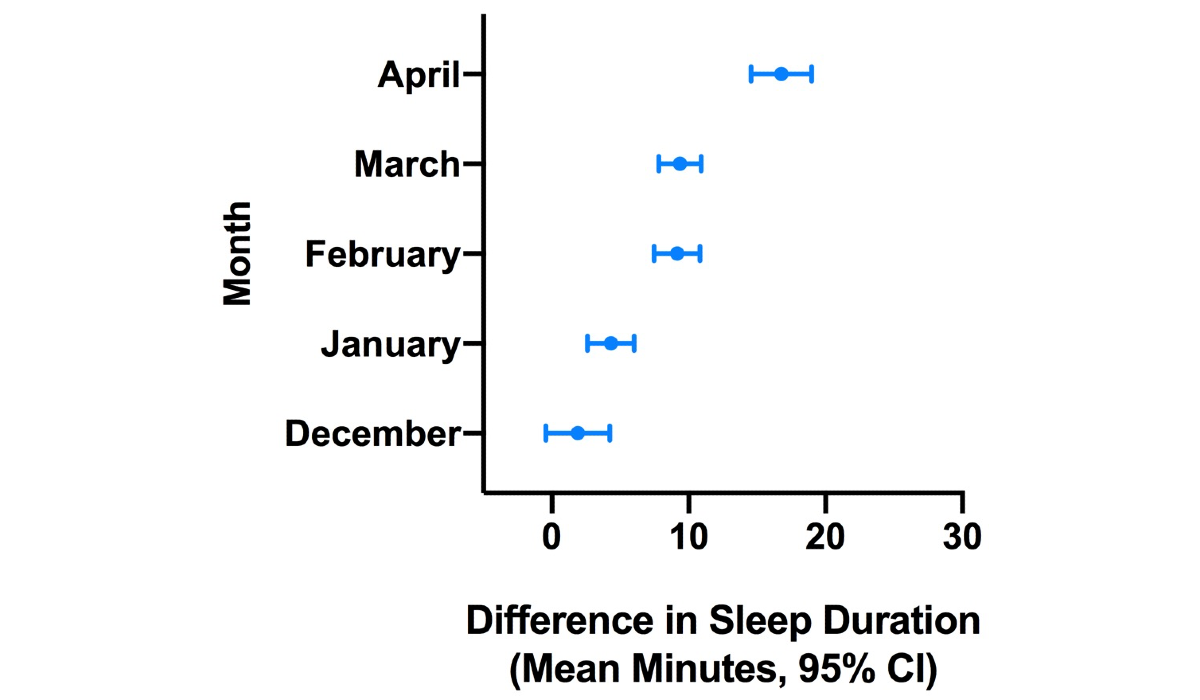
Difference in estimated sleep duration during the COVID-19 pandemic compared to the same month in the previous year in Stockholm.

Results from the generalized linear mixed models are displayed in Figures 3-8. As shown in Figure 3, estimated sleep duration increased after the onset of COVID-19 in the total sample. In the total sample, comparing January 2019 to January 2020, we observed increases of 4.9 minutes of estimated sleep duration (95% CI 4.3-5.5 minutes; *P*<.001); comparing February 2019 to February 2020, we observed increases of 5.2 minutes of estimated sleep duration (95% CI 4.81-5.91 minutes; *P*<.001); comparing March 2019 to March 2020, we observed increases of 13.7 minutes of estimated sleep duration (95% CI 13.1-14.3 minutes; *P*<.001); and when comparing April 2019 to April 2020, we observed increases of 22.3 minutes of estimated sleep duration (95% CI 21.5-23.2 minutes; *P*<.001) (Figure 3).

Models examining estimated sleep duration after the COVID-19 outbreak by city are displayed in Figures 4-8. Seoul had the smallest increase when comparing April 2019 and April 2020 (12.2 minutes, 95% CI 9.5-14.4 minutes; *P*<.001), while New York had the largest (24.5 minutes, 95% CI 23.1-25.9 minutes, *P*<.001), followed by London (20.8 minutes, 95% CI 19.5-22.1 minutes; *P*<.001).

## Discussion

### Principal Findings

The results of this study, based on 2.9 million nightly recordings of smartphone-estimated sleep duration, demonstrate an abrupt, significant increase in estimated sleep duration in the months after COVID-19 was declared a pandemic and international health crisis by the World Health Organization [17] compared with the same months from the same individuals in the prior year. Further, the average estimated sleep duration in several regions was at or below the lower range of the recommendation for healthy sleep before the pandemic (ie, 7 hours or less), even among those concerned about sleep enough to track it; however, this duration increased to be within the range of healthy, sufficient sleep amid the onset of the COVID-19 pandemic. Our data, gathered from a daily sleep tracker smartphone app, reveal that on average there were significant increases in estimated sleep duration in London, Los Angeles, New York City, Seoul, and Stockholm concomitant with the onset of the COVID-19 pandemic, consistent with data from subjective, retrospective self-reports among 425 adults in 3 European countries [18]. We hypothesize that these increases in smartphone app–estimated sleep duration, together with the recently reported increases in sleep and wake time regularity following the onset of the COVID-19 pandemic [19], have occurred as a consequence of stay-at-home orders, nonessential business closures, and work-from-home policies implemented to slow community spread of the infection. Sufficient sleep duration is a critical element of immune system functioning, with inadequate sleep resulting in increased susceptibility to viral infection and reduced antibody production after vaccination [8,9]. We cannot exclude the possibility that individuals in this study were experiencing longer times in bed as opposed to longer estimated sleep duration. Nevertheless, if the observed increase in estimated sleep duration, as detected by the app, in fact represents an increase in actual sleep amid the COVID-19 pandemic, this may have been an important factor in reducing the risk of progression to COVID-19 following exposure to SARS-CoV-2 [10,11] and in enhancing the ability of people with COVID-19 to mount an effective immune response [12,13].

Additionally, we detected notable differences in sleep by geographic region. The average estimated sleep duration prior to the COVID-19 pandemic of app users in Seoul was >1 hour shorter than those in London and Stockholm, which is consistent with prior research, and >40 minutes shorter than those in New York City and Los Angeles. This is consistent with prior research, which documented that sleep deprivation is common in South Korea [20,21]. Moreover, app users in Seoul also demonstrated the smallest increase in estimated sleep duration during the COVID-19 pandemic, at 11 minutes per night compared to the increases of 20 minutes in other cities. The smaller increase in Seoul compared to other cities could be related to the outbreak of SARS in South Korea in 2003, which may have afforded an opportunity for the country to prepare and contain the spread of the virus without the more drastic mitigation measures, such as extended stay-at-home regulations, that were levied in other countries. Further, as the increase was very small, it also may be due to a natural month-to-month variation as opposed to trending in the direction of increased sleep duration as observed in the other cities. Although Sweden did not initially institute the stringent COVID-19 containment measures other countries chose to enact (eg, shelter-in-place policies), the estimated sleep duration increased in Stockholm at a rate similar to that in other regions that did institute stringent containment policies at the time. This may reflect that people residing in Stockholm are following precautions to another people worldwide, despite not being restricted to their homes. Finally, the observed increase in estimated sleep duration in New York City of >20 minutes could be explained in part by the high number of cases of COVID-19 in New York City and the associated strict stay-at-home policies.

The increase in the estimated sleep duration observed in this study in response to the COVID-19 pandemic of between 12 and 24 minutes is of similar magnitude to the approximately 25-minute increase in total sleep time induced by controlled-release zolpidem [22]. Thus, the COVID-19 mitigation policies are associated with an increase in estimated sleep duration comparable to the impact of a hypnotic medication. This may be clinically important, as sleep deficiency—which is highly prevalent in developed countries—increases susceptibility to viral infection and diminishes the immune response to vaccination [10,11].

Although we detected an increase in estimated sleep duration after the onset of COVID-19 in several different metropolitan areas, the amount of sleep disturbance may have changed after the onset of the pandemic. Previous research has shown that sleep quality suffers in the face of major crises [2]. Specifically, research has documented a significant increase in sleep difficulty and arousal from sleep amid the 2003 SARS outbreak [4], during the bombings in Israel during the Persian Gulf War [3], and in the aftermath of natural disasters [5,6], consistent with the reported increase in self-reported insomnia symptoms in Wuhan, China, at the start of the COVID-19 pandemic [23]. Our data indicating an increase in the estimated sleep duration do not preclude the possibility that more people were suffering from disturbed sleep. Additional studies administering validated questionnaires assessing insomnia symptoms as well as self-reported sleep quality would be required to address this question.

### Limitations and Future Research

This study is subject to a number of limitations. (1) Although a strength of our study is the large number of respondents offering user-generated sleep data over time and across several geographic regions, a limitation is that we did not obtain data pertaining to sleep latency, awakenings from sleep, or time in bed after waking. (2) Although users do not commonly use the app for napping, we removed observations that were shorter than 60 minutes. Future research may examine naps and how napping behaviors changed due to the COVID-19 pandemic and its mitigation strategies. Further, we did not have access to sleep timing data, which would have been useful in understanding whether users were going to sleep earlier or sleeping later as a result of the COVID-19 pandemic. (3) Employment or income demographics, which are not collected by Sleep Cycle, may represent unmeasured, confounding variables in our study. For instance, individuals from higher socioeconomic groups were more likely to remain employed and to work from home during the COVID-19 pandemic [24]. Those who were furloughed or remained employed and were allowed to work from home may have had more flexible work hours and spent significantly less time commuting, allowing them to spend more time in bed [25]. Moreover, with the proliferation of low-cost delivery services (eg, food and other goods) in major urban centers, users who were able to work from home may have had increased available time for sleep [26]. These results may therefore not be generalizable to those working essential jobs, whose work hours and commuting time may have increased during the COVID-19 pandemic and who may be underrepresented in this sample. (4) The sleep tracker smartphone app is not validated, and because it does not account for waking after sleep onset, it is possible that the tracker overestimates sleep duration. (5) Those who download and use the sleep tracker smartphone app consistently may be more interested in sleep and health in general, which may represent selection bias, survivorship bias, and less generalizability to the general public.

Our study identifies several avenues for future research; some reflect the limitations of the app. First, it is important to explore whether the findings we observed are transient or will persist for an extended period and whether other aspects of sleep changed during the COVID-19 pandemic. Second, data from the Sleep Cycle app require validation using objective measures, such as polysomnography or actigraphy. Third, it would be informative to determine the prevalence of sleep disturbance or insomnia symptoms, such as difficulty falling asleep or difficulty maintaining sleep, among users of this app. Finally, an exploratory qualitative investigation with users of Sleep Cycle may help to determine if actual sleep duration in fact increased during the COVID-19 pandemic or if users experienced more sleep difficulties despite having increased time in bed.

### Conclusion

Public health officials' policies to mitigate the COVID-19 pandemic have required profound changes in peoples' daily routines worldwide. Using approximately 2.9 million nights of objectively recorded sleep on 3 continents via a sleep tracking smartphone app, we observed an abrupt, significant increase in average estimated sleep duration among 8218 unique users in 5 cities in the months after the onset of the COVID-19 pandemic. Thus, not only have COVID-19 mitigation strategies reduced the potential of infection with SARS-CoV-2, but the resulting increase in sleep episode duration may also have improved the ability of the immune system to resist infection. Future research should explore whether this observation is transient or persists over the long term and whether other aspects of sleep have changed amid the COVID-19 pandemic.

## Abbreviations

BWH: Brigham and Women’s Hospital
NIOSH: National Institute for Occupational Safety and Health
SARS: severe acute respiratory syndrome
