# Correction: Estimated Sleep Duration Before and During the COVID-19 Pandemic in Major Metropolitan Areas on Different Continents: Observational Study of Smartphone App Data

**DOI:** 10.2196/28057

**Published:** 2021-02-22

**Authors:** Rebecca Robbins, Mahmoud Affouf, Matthew D Weaver, Mark É Czeisler, Laura K Barger, Stuart F Quan, Charles A Czeisler

**Affiliations:** 1 Division of Sleep and Circadian Disorders Departments of Medicine and Neurology Brigham and Women's Hospital Boston, MA United States; 2 Division of Sleep Medicine Harvard Medical School Boston, MA United States; 3 Department of Mathematics Kean University Union, NJ United States; 4 School of Psychological Sciences Turner Institute Brain and Mental Health Monash University Victoria Australia; 5 Institute for Breathing and Sleep Austin Health Melbourne Australia

In “Estimated Sleep Duration Before and During the COVID-19 Pandemic in Major Metropolitan Areas on Different Continents: Observational Study of Smartphone App Data” (J Med Internet Res 2021;23(2):e20546) the authors noted one error.

In the originally published paper, the name of one author cited in Reference 25 (Czeisler MÉ) was incomplete. The list of authors cited in Reference 25 originally appeared as follows:

Czeisler M, Howard ME, Robbins R, Barger LK, Facer-Childs ER, Rajaratnam SM, et al.

In the corrected version of the paper, the list of authors appears as follows:

Czeisler MÉ, Howard ME, Robbins R, Barger LK, Facer-Childs ER, Rajaratnam SM, et al.

The correction will appear in the online version of the paper on the JMIR Publications website on February 22, 2021, together with the publication of this correction notice. Because this was made after submission to PubMed, PubMed Central, and other full-text repositories, the corrected article has also been resubmitted to those repositories.

